# Challenges in physician supply planning: the case of Belgium

**DOI:** 10.1186/1478-4491-8-28

**Published:** 2010-12-08

**Authors:** Sabine Stordeur, Christian Léonard

**Affiliations:** 1 Belgian Health Care Knowledge Centre (KCE), Administrative Centre Botanique, Doorbuilding (10th floor), Boulevard du Jardin Botanique 55, B-1000 Brussels, Belgium

## Abstract

**Introduction:**

Planning human resources for health (HRH) is a complex process for policy-makers and, as a result, many countries worldwide swing from surplus to shortage. In-depth case studies can help appraising the challenges encountered and the solutions implemented. This paper has two objectives: to identify the key challenges in HRH planning in Belgium and to formulate recommendations for an effective HRH planning, on the basis of the Belgian case study and lessons drawn from an international benchmarking.

**Case description:**

In Belgium, a numerus clausus set up in 1997 and effective in 2004, aims to limit the total number of physicians working in the curative sector. The assumption of a positive relationship between physician densities and health care utilization was a major argument in favor of medical supply restrictions. This new regulation did not improve recurrent challenges such as specialty imbalances, with uncovered needs particularly among general practitioners, and geographical maldistribution. New difficulties also emerged. In particular, limiting national training of HRH turned out to be ineffective within the open European workforce market. The lack of integration of policies affecting HRH was noteworthy. We described in the paper what strategies were developed to address those challenges in Belgium and in neighboring countries.

**Discussion and evaluation:**

Planning the medical workforce involves determining the numbers, mix, and distribution of health providers that will be required at some identified future point in time. To succeed in their task, health policy planners have to take a broader perspective on the healthcare system. Focusing on numbers is too restrictive and adopting innovative policies learned from benchmarking without integration and coordination is unfruitful. Evolving towards a strategic planning is essential to control the effects of the complex factors impacting on human resources. This evolution requires an effective monitoring of all key factors affecting supply and demand, a dynamic approach, and a system-level perspective, considering all healthcare professionals, and integrating manpower planning with workforce development.

**Conclusion:**

To engage in an evidence-based action, policy-makers need a global manpower picture, from their own country and abroad, as well as reliable and comparable manpower databases allowing proper analysis and planning of the workforce.

## Introduction

The healthcare sector is labor intensive and human resources represent the most important input into the provision of health care, as well as the largest proportion of health care expenditure [1,2]. Reliable planning of human resources for health (HRH) is therefore crucial. It is the process of projecting the required health workforce to meet future health service demand and the development of strategies to meet those requirements [3,4]. Processes and means to attain such an objective are far from simple however, as fundamental societal and institutional factors impact upon health workforce production [5]. Therefore, policy planners worldwide are recurrently faced with the question of the appropriate number of health professionals needed given population health needs and trends in health service utilization and production. To address this question, a number of forecasting tools have been put forward. Nowadays, many countries, such as Belgium, Canada, France, the United Kingdom and the United States, are balancing from projections of surplus to warnings of shortage with a perplexing frequency [6]. At least two factors can explain this development. On the one hand, forecasting tools might be deficient and need to be refined, as we have recently highlighted [6]. On the other hand, policies might be inadequate or inadequately implemented. We propose to examine the latter hypothesis with the means of a case study of Belgium.

In Belgium, medical training consists of a seven-year university course, divided into two cycles: the Bachelor's degree (three years) and the Master's training (four years). Once graduated, physicians need a practice license which is granted by the Federal Ministry of Public Health. Further training is needed to obtain this license. Medical graduates wishing to become specialists are further trained during two to six additional years, according to their specialty. There are 30 recognized specialties including general practice. After having obtained their official license, new graduates are allowed to register with the National Health Fund.

The objective of this paper is two-fold. First, four key challenges in health workforce planning in Belgium will be outlined: 1) projecting the right physician supply, 2) tackling specialty imbalances, 3) dealing with geographical imbalances and 4) apprehending international mobility. Second, for each key challenge, we will identify solutions applied in Belgium as lessons drawn from other countries to formulate recommendations for an effective health workforce planning.

## Methods

For mapping the current policy of physician supply planning in Belgium, we used 3 main sources of information. First, we reviewed all relevant legal texts published between 1996 and 2009 in order to assess policies and institutional mechanisms in relation to workforce supply. Second, selected stakeholders (members of the Committee of Medical Supply Planning; members of the Ministry of Public Health and The European Social Observatory; and members of the National Institute for Health and Disability Insurance) were interviewed for additional insights on specific aspects of the HRH policy. Third, we benchmarked the Belgian case against a number of neighboring countries which display diverse educational policies and regulations, specifically France, Austria, Germany and the Netherlands. Data collection for the benchmarking was based on a literature review, including grey literature such as reports from international or national institutions, and on consulting stakeholders in each of the countries.

## Case description

### Setting the right physician supply

#### Adopting a restriction mechanism

In the mid-1990 s, Belgium had the highest physician to population ratio in industrialized countries (35 physicians per 10 000 inhabitants in 1995). The assumption of a positive relationship between physician density and health care utilization was a major argument in favor of medical supply restrictions [7]. Secondary motivation concerned the quality of care. In a context of potential oversupply a low number of contacts with patients may interfere with the quality of care [8]. Moreover, important variations between Regions (i.e. a higher ratio in the southern region, Wallonia, compared to the northern region, Flanders) were considered neither politically acceptable nor financially sustainable given the federal financing of health care. Consequently, the Federal Government decided in 1997 upon a *numerus clausus *to limit the total number of physicians working in the curative sector and to gradually smoothen the existing discrepancy in the medical density between the regions.

Practically, the restriction mechanism applies at the end of the core training (seven years) and limits the number of trainees allowed to specialize as general practitioners (GP) or medical specialists (SP). Thus, since 2004, not all medical graduates were allowed to register with the National Health Fund. The number of practice licenses was set at 700 for the year 2004, 757 for the years 2008-2011, 890 for 2012, 975 for 2013, 1 025 for 2014 and 1 230 for the years 2015-2018 (in comparison to approximately 1 200 licenses delivered in 1999). Some specific specializations, i.e. data management, forensic medicine and occupational medicine, fall outside the overall quota ('out of quotas') as these are not financed through the National Health Fund.

These licenses are split over the regions in proportion to population size (60% for Flanders; 40% for Wallonia), as well as between general practitioners (43%) and specialists (57%). The apportionment basis between regions was decided whereas the health needs of the two regions have not been studied, e.g. it was demonstrated that citizens of the southern region had a shorter life and a shorter healthy life than their northern counterparts [9]. Moreover, the productivity of Walloon practitioners working in curative health care is estimated lower than that of their Flemish colleagues [8].

Officially registered physicians are estimated to give a medical density of 42.2 per 10 000 inhabitants in 2005 [10]. However, only a proportion of registered physicians is active, and an even smaller proportion is active in the curative sector. For example, Roberfroid et al. (2008) [11] showed that in 2005, only 53.3% (11 626/21 804; 11 per 10 000 population) of all GPs and 65.4% (13 328/20372; 13 per 10 000 population) of all specialists were practicing physicians. This report estimated that one fifth to one third of active physicians worked in other fields of activity than curative care, such as scientific research, administrative service, employment in pharmaceutical companies and insurances. The overall density of practicing physicians is then in reality between 23.8 and 28.1 per 10 000 inhabitants [11]. The report also highlights a potential attrition in the number of practicing GPs, its quantity decreasing from 12531 in 2002 to 11626 in 2005 (-7%). Moreover, in 2005, 47.7% of practicing GPs and 45.6% of practicing specialists were older than 50 years and 30.1% of the medical workforce were women. This latter proportion increased to 59.5% in new graduates [11]. This phenomenon might have an increasing impact on the future workforce since women are more likely to take career breaks or to work part-time. Lorant et al. (2008) also emphasized the growing part of inactive doctors, 19% of all registered GPs in 2000 being inactive in the healthcare sector 5 years later (in 2005). The part of inactive GPs was more important among women (21%) than men (15%) [12].

To address a possible future shortage of GPs, the above ratio of 43/57 between GPs and SPs had to be revised. Since 2009, the numbers for GPs were not maxima to respect but minima to be reached: an annual minimum of 300 GPs have to be trained during 2008-2014 and 360 during 2015-2018. For specialists, the rule of maxima has to be maintained except for 3 specializations, for which a shortage is already observed: child psychiatry, acute medicine and emergency medicine. A minimal number of such specialists has to be trained and registered annually to maintain a sufficient stock of physicians in the specialties.

#### Fulfilling the quotas

The Federal Minister of Public Health fixes the number of practice licenses available to trainees every year. However, the Community Ministers of Education bear the responsibility to adapt students' intake such that it fits the number of trainees who will be eventually allowed to further specialize as GPs or SPs (the Flemish Community exercises its competences only for Dutch speaking people (which coincides with Flemish Region in the North - or Flanders - and a part of the Brussels-Capital Region) and the French Community for French speaking people (which coincides with Wallonia in the South and the other part of the Brussels-Capital Region)). The way the numerus clausus was implemented in universities varies by community. The Flemish community introduced an entrance examination (i.e. everyone passing the exam is eligible to register in university training) while the French community opted for a selection procedure after the first year, with only a percentage of the successful students being selected.

During the period 2004-2008, there were 282 fewer registered doctors than the number authorized. So, the federal objective of restricting the number was reached. However, the repartition between communities was uneven: the Flemish Community was under its legal quota during this period (minus 319 doctors) whereas the French Community was beyond its quota (plus 37 doctors).

Conversely, for the following years, the expected number of registered physicians, as estimated on the basis of the current number of students' inscriptions, will exceed the authorized quotas. In 2011, 7 years after the implementation of the numerus clausus, there will be an excess of more or less 400 graduates (220 in the Flemish Community and 180 in the French Community), due to higher numbers of candidates who pass the entrance exam in the Flemish Community and a relaxing of restriction policy in the French Community. These graduates will either have to opt for an 'out-of-quota' specialty, or begin their specialization in a foreign country, or choose a professional activity not requiring a license (e.g. researcher, teacher or civil servant). During the period 2004-2012, 468 applicants (281 in the Flemish Community and 187 in the French Community) can choose one of the three specialties where minimal numbers to be trained were defined.

In 2008, in the French Community, the Belgian Court of Justice eventually acknowledged the illegality of not authorizing students who successfully ended their first year in a medical faculty to pursue their training. Consequently, The Minister of Higher Education in the French Community unilaterally decided to (temporarily) stop restricting student access to the full medical cursus, whereas the Flemish Community maintained its selection procedure. Moreover, the restriction is still valid at the federal level and it is unclear how the supernumerary students will legally practice.

#### Specialty imbalances

During the period 2004-2008, a 19% oversupply of specialists was recorded whereas 25% of the GP quotas were unfulfilled. This phenomenon is more acute and worsening in Flanders, where the actual inflow of GPs was 344 units lower than the requirements, compared to only 75 units in Wallonia.

One strategy to increase the attractiveness of general practice has been to increase the exposure to primary care experiences during residency. In recent years, the development of Academic Centers of General Medicine has given general medicine more visibility to candidates. A profound reform of the medical training program was implemented. From the very beginning of the Master studies, students benefit from specific courses as a training period in general practice. This permits, in their second phase of medical studies, to have a better vision of this practice. Lecture courses for large audiences were abandoned in favor of interactive lessons for small groups, enriched by personal feedback from student's experiences in GP's practices. The more pro-active academic centers in the French Community register higher rates of inscription in GP training. This may explain the lower GPs shortage in Wallonia as compared to Flanders.

Specialties such as child psychiatry, acute medicine or emergency medicine are also considered short of candidates. Specialties offering a more regular work schedule, more leisure time and higher earnings are increasingly chosen, reflecting a desire among physicians to balance professional life and social commitments [13]. To counter this phenomenon, minimum numbers of positions in these medical specialties that should be annually filled were defined in order to guarantee a sufficient renewal of the stock. This new regulation was really successful during the period 2004-2008 as these three specialties recorded 26% more inscriptions than defined by the minima.

#### Geographical distribution of medical practitioners

In Belgium, physicians can freely choose their practice location. This results in geographical imbalances in physician density. The density of practicing GPs varies between provinces from 9.8 GPs to 14.4 GPs per 10000 inhabitants. The density of practicing SPs also varies between provinces from 8.4 SPs in rural areas to 24.0 SPs per 10 000 inhabitants in Brussels. The higher density of SPs in big cities relates to the higher number of hospital beds and the proximity of specialized hospitals. As in other countries, physicians are more likely to settle and practice in affluent, metropolitan areas than in rural areas.

As geographical imbalances may generate inequity in health care accessibility, the challenge is to achieve a more even distribution of practitioners. To counterbalance these, an 'attraction policy' has been recently implemented. Since 1 July 2006, a specific fund (Impulseo I) was proposed to encourage general practitioners to settle down in areas which have a low physician density, i.e. less than 9 GPs per 10 000 inhabitants, or in areas with less than 12 GPs per 10 000 inhabitants, and less than 125 inhabitants per square kilometer. This provision is also offered to encourage GPs to settle in areas qualified as 'positive action areas' within the political framework for big cities (precariousness). The Impulseo package includes: a premium of €20 000, €15 000 for interest-free loan, €30000 for additional loan, and free administrative assistance during the first 18 months of the installation. In 2007, 205 of the 589 official municipalities (35%) were recognized as a target zone for Impulseo I. Between 2006 and 2008, 352 GPs have received financial support to install in rural areas.

However, the low numbers of physicians in rural areas have not solely to do with recruitment but also with physicians' preferences, as heavy workloads, lack of equipment and supplies, and of appropriate facilities lead doctors to look for better working conditions [14]. Another incentive to attract and retain physicians in a specific areas is encouraging group practice to favour teamwork and facilitate planning and sharing out of duty hours. A second specific fund (Impulseo II) was proposed to financially support GPs in employing an administrative coordinator in the context of group practice and management of the patient's electronic file. Since 2007, 1 260 GPs benefited from this specific support (330 registration forms for duo practices, 225 for trio practices, 129 for practices with 4 doctors, and finally, 69 for bigger practices), for a global amount of €6.6 millions. Of all requests, 77% come from Flanders, 16% from Wallonia and 7% from Brussels Capital. The higher rate recorded in Flanders is possibly a reaction to the higher GP shortage in this Region. However, this difference is not yet deeply evaluated.

This project could be extended to GPs having at least 150 patients' electronic files and working in solo, in order to relieve their administrative workload (Impulseo III). Moreover, new pilot projects to ensure the continuity of care in such areas are currently being tested. The more important one is a call-centre that centralizes and dispatches calls towards more appropriate services (emergency service or general practitioner). The main objective is to reduce home visits by adequately addressing patients. Other objectives are increasing the security for GPs and recording the frequency of calls, i.e. by region and by period (day/night, week/week-end).

#### International mobility of students and practitioners

Medical supply planning has remained a national responsibility while European regulations, including those impacting on medical supply planning, have become mandatory for member states. In particular, professionals have the right to settle and to provide medical services anywhere in the EU (the so-called 'physicians directive', passed in 1993).

Since 2004, the number of foreign physicians licensed to practice in Belgium has sharply increased. New visas delivered annually to foreign medical doctors rose from 78 before 2004 to 430 in 2008. Before 2004, the inflow originated largely from the neighboring countries (France, the Netherlands and Germany) and to a lesser extent from Spain and Italy. Since 2004, the largest group of immigrant doctors comes from the Eastern part of the European Union (Poland and Romania).

As the physicians' directive also applies to students, those originating from countries with a restricted access to medical training are keen to search for training opportunities in other European countries. In Belgium, numerus clausus only applies for medical doctors with a Belgian diploma. The number of foreigners specializing in Belgium as a GP or specialist has increased from 38 in 2004 to 78 in 2006, i.e. 4.4% and 10.4% of all trainees, respectively. Between 2004 and 2006, only 3% of all foreign students came to Belgium to obtain a GP diploma, whereas the majority of these students (97%) opted for a specialization whose access is restricted by quotas to Belgian doctors. None opted for an out-of-quota specialization. Preferred options were anesthesia-reanimation, surgery and pediatrics. Meanwhile, in 2007, more or less 400 doctors with a Belgian visa left the country just after obtaining their specialization.

Belgium has attempted several times to avoid the massive influx of foreign students. In 1971, the French Community of Belgium declared that foreign applicants for medical studies ought to qualify for medical studies in their own country or to pass an aptitude test which was not required from holders of a Belgian secondary education diploma. This was considered to be discriminatory by the European Court of Justice. Consequently, in 2003, the Belgian French Community specified that the rule did not apply to citizens of another European Member State [15]. Since 2006, the French Community of Belgium established a 70% quota for students residing in Belgium, to react against a massive inflow from France. The European Commission opined that this measure was not justifiable. Belgium abolished this discriminatory system and put in place a new one. In November 2007, the Commission officially decided to suspend the infringement case against Belgium [15], acknowledging that without this restrictive measure, a problem could arise in the future for the quality and the sustainability of the Belgian health system. Nevertheless, Belgium has to submit supplementary data within 5 years to justify the necessity and the proportionality of this measure. The restriction imposed by the French Community seems to have no effect for medical specializations.

These international flows of medical personnel make any planning exercise of national health professionals' supply quite difficult. It should also be noted that the phenomenon is poorly documented so far. Only crude data are available, and important parameters such as the proportion of immigrants getting the practice license for training reasons (specialization) who will stay in Belgium, turnover rates or activity profiles, are currently unknown [6].

### Lessons learned from the international comparison

#### Setting the right physician supply

France, Belgium, Germany and the Netherlands have implemented a numerus clausus, while in Austria the access to medical studies is not restricted by any quotas. The numerus clausus is made effective in controlling the intake of medical students through either a competitive entrance exam or, in the case of France, in controlling the number of students entering the second year of study in medical schools, as in the French Community of Belgium. In the Netherlands, students are selected by lottery.

The objective remains limiting the students' intake in Belgium and Germany. In Germany, a revised set of licensing regulations was introduced in 2002, implying a statutory reduction of up to 10% in the number of available places for studying medicine [16]. France and the Netherlands, following a perceived medical workforce undersupply, tended to reverse the situation by increasing the students' inflow. The recent history of these two countries demonstrates the difficulty of reaching and keeping an appropriate medical workforce. As in France, the diagnosis of undersupply can sometimes within a few years turn to oversupply. Several factors can explain this. The main one is probably that appropriate numbers are determined by relatively crude forecasting methods. These methods aim to assess the current stock and its likely demographic changes and to estimate the future demand for health care. Demand forecasts are mainly based on demographic changes in the population; more recently, they tend to include epidemiological or system-wide changes. Another factor relates to the important time lag involved in training medical students (12-13 years for some specialties). After such a period, the health care system and its suppliers may have drastically changed and supply may no longer match demand.

#### Specialty imbalances

In all studied countries, specialists outnumber GPs. While general practice is mainly appreciated for its variety in work, autonomy and the privilege of working with patients in different stages of their life, it is not as attractive as medical specialties. This lack of attractiveness is explained by predominantly curative and specialized care, a hospital-centered model of medical education with little experience of primary care, the lack of prestige, lower income levels, a heavy workload, a lot of uncertainties during the clinical decision making process, the absence of teamwork, and the insufficient intellectual content [12,17].

In France, 1 000 training posts in general practice remained unfilled in 2005, i.e. 41.7% of all available posts [18]. Specialties such as gynecology or pediatric were also undersupplied [19], and some were even fading out, e.g. neuro-psychiatry, radiology and medical imaging [20]. This situation has led the government to adopt a first strategy, i.e. implementing national ranking examinations [21] in order to regulate the number of physicians by specialty. The implementation of these national tests led to a change in the distribution of generalists/specialists (targeted at 50/50) in the choice of junior doctors' posts, which had been less than 40% for general practitioners [17]. However, this system did not succeed in regulating repartition of students between specialties, as the overall number of available positions for all specialties has always outweighed student numbers. A second strategy was based on increasing exposure to primary care experiences during the whole medical training. In France, medical students now benefit from an obligatory two-month training period in general practice [17,22].

Measures providing incentives to choose certain disciplines are adopted as a third strategy. For example, in France, study grants are proposed to students undertaking specific training courses or a period of general medicine. On the basis of these experiments which involved very few doctors, it seems that the costs incurred are an obstacle to their generalization [17]. In Germany, half of the GP-trainees' salaries during the office based training period (minimum two out of five years) is publicly financed. However, in practice, the subsidy is often the trainee's income only, which may explain that attractiveness remains quite low [23].

To counter GPs shortage, some countries also introduced change in healthcare workforce skill-mix. Changes in the skill-mix may affect the workload as well as the number of physicians required. Since 2005 France has developed pilot projects transferring some specific tasks from physicians to other professional categories. For example, management of dialysis is delegated to nurses and the prescription of eye glasses to orthoptists. The law has also been adapted to authorize drug prescriptions by nurses. Ten new experiments look at the delegation of the follow-up of chronic patients to non-medical practitioners [24]. An evaluation of these experiments is currently underway. In the Netherlands, the Nurse Practitioner (NP) was invented at the end of 1997, originally to meet several human resource problems: a shortage of physicians, the need for continuity and coordination between patients and healthcare workers, and the lack of career possibilities for nurses. It turned out that NPs endorse tasks which were previously neglected by GPs (e.g. prevention, education and controls). Consequently, although contributing to quality of patient care, they neither alleviate GPs workload nor replace them [25]. A national experiment is currently underway concerning the extension of primary care tasks for nurses. Within this program, qualified nurse practitioners can visit patients at home, care for patients with chronic conditions (asthma, arterial hypertension, diabetes, etc.) and manage vaccination programs. However, they may not make any diagnoses or issue prescriptions.

Before implementing such innovations, appropriate HRH planning is necessary, as well as continuous training to develop skills and knowledge of collaborators. Task delegation from doctors to nurses, leaving doctors to manage the more complex patient problems while delegating care to nurses, can lead to an excessive workload for nurses unless their numbers are increased and/or simpler tasks are delegated to nurse auxiliaries or health care assistants [26,27].

#### Geographical imbalances

Policies regulating the national supply of physicians do not necessarily influence the geographical distribution of doctors [14]. Therefore, countries implemented one or a number of complementary policies designed to even out the geographical distribution of the medical workforce.

Two main policy options have been considered to address the problem, i.e. an incentive-based versus a directive approach. France as well as Belgium adopted the first strategy, which was implemented through various components. First, public authorities allocated a relatively higher inflow of medical students to underserved regions. However, a circular stipulating that the number of work positions must be significantly higher than the number of students did counter the policies in place for the last few years in certain regions. Moreover, specialists may find internships in regions where there are fewer doctors ('forced migration') and then return to their region of origin to practice [28]. More positive results in the long term were reported for policies designed to reform basic training, including: a significant rural experience in the training curriculum; particular attention to students living in rural areas and who are likely be located in underprivileged areas in the future; or measures to adapt the content of training to the skills needed in these areas [29].

Secondly, in France as in Belgium, plans were introduced to encourage doctors to practice in medically deprived areas. In France, municipalities can provide financial aid to doctors who wish to set up a practice in deprived areas, allow tax exemptions or provide them with professional facilities or personal housing. They can also give a study allowance, offer a housing grant or provide accommodation to medical students in their sixth year of study if these students commit to living for a minimum period of five years in a medically deprived area. Social security also plays a role in the installation of doctors' practices, by offering regional information tools, helping candidates to visualize the healthcare offer and activity within a given region ('CartoS@nte') and providing information on the funding and assistance available ('InstalS@nté'). In practice, the assistance and monitoring provided by the local social security offices is not particularly well developed [17]. Since 2005, National Social Security has also implemented good practice contracts in order to encourage the installation or maintenance of general practitioners in specific zones (mountain resorts, urban free areas or rural zones). However, at the end of 2005, these measures had attracted very few doctors, since the take-up rate is systematically less than 10% [24].

Third, encouraging group practice was also an option. In France, a special fund (Fund to sustain Quality of Care in Cities ('Fonds d'Aide à la Qualité des Soins de Ville)) within the national health insurance budget, can be used to make capital investments to set up multi-specialty group practices. Additionally, a new status of 'associated partner' has been created for young doctors. This will allow them to join a practice without having to make a capital investment.

Fourth, strategies to sustain health professionals working in rural areas encompass new technologies such as tele-health and telemedicine, facilitating professional collaboration and development by supporting, for example, continuous education and access to services (interpretation of x-rays, specialist opinions) [14]. It is noteworthy that an evaluation of these policies is lacking so far. The costs incurred are often unknown and could be an obstacle to generalizing experiments such as this.

Whatever the measures adopted, it is absolutely necessary to coordinate the measures, stakeholders and institutions involved in order to ensure that human resources are distributed in a way that meets the needs of local populations [17]. The French examples highlighted the negative effects of contradictive policies.

Countries such as Germany and Austria have adopted a regulatory policy that imposes conditions on the choice of practice location. Physicians are not able to get a contract with a regional health insurance fund if the threshold number of physicians is reached in that region. For instance, in Germany, since 1993, new practices may not be opened in areas where supply exceeds 110% of the defined threshold, thresholds being based on the physician-to-population ratio of 1990 [16,30]. Although there is almost no possibility to establish new practices for specialists in Germany, general practitioners are free to set up their own practice in two-thirds of the country, mainly in the eastern part of the country. In both countries, the geographical distribution of doctors has become more even. Still, this policy has its own shortcomings. First, the existing oversupply in large cities was not resolved since there was no instrument for closing practices or preventing others from taking over the practices and registrations of retiring doctors. Second, it was seen as partly responsible for the attrition of medical students during training and the subsequent decrease in the number of new graduates [16].

#### International mobility

Having no cap on student numbers, Austria faces a particular challenge. To avoid a massive influx of German students into its medical faculties, Austrian law made holders of secondary education degrees acquired in other European Member States, seeking access to higher education in Austria, subject to additional conditions to satisfy the general Austrian requirements for access to higher or university education. Austria invoked the interest in safeguarding the homogeneity of the Austrian education system [15]. Moreover, Austria feared that a massive influx of German students would endanger the Austrian health system leading to a shortage of doctors since these students would return to Germany after having completed their studies. An amendment similar to the Belgian example was installed. Following the European Court's decision, Austria provisionally amended the relevant Universities Act twice, firstly in July 2005 to abide by the Court's decision, and once more in June 2006, to re-establish restrictions to the access to Austrian universities. The latter amendment specified that, for some courses of studies, 75% of the study places should be reserved to applicants with a secondary education diploma acquired in Austria, while a further 20% should be reserved for other EU students, and the remaining 5% to third-countries students. As in the case of Belgium, the Commission officially decided to suspend the infringement case against Austria and required supplementary data within 5 years to justify the necessity and the proportionality of the measure implemented [15].

In France, the Netherlands and Germany, increasing immigration of medical practitioners is seen as a means to maintain an adequate stock of physicians [31]. Foreign-trained physicians make a substantial contribution to the physician workforce where a shortage of medical workforce is observed. For example, in Germany, where there are important imbalances between geographical areas, with the lowest physicians' density in the eastern states, more hospitals look abroad for doctors, particularly in Eastern Europe. International recruitment campaigns are particularly active, involving advertisements in the medical press and participation in job fairs in Germany. The most common countries of origin are Greece, Iran, Poland, the former Soviet Union, Syria and Turkey,

## Discussion and evaluation

As far as medical supply in Belgium is concerned, the preceding analysis brings up a number of issues. First, there are considerably fewer practicing than registered physicians and the practicing physicians' density decreased significantly over time. This decrease might have resulted from an important professional attrition rate [32,33]. Consequently, different indicators lead to a fear of future shortages, particularly among general practitioners: the decreasing productivity in young cohorts of registered doctors, the increasing proportion of active doctors who stop their curative activity before their retirement age and, finally, the retirement of substantial cohorts of graduates in the years 2015-2025 [8]. Moreover, for the whole medical workforce, new regulations such as the European ones aiming to limit the working hours of young specialists in training are changing the working perspective. These considerations were partly taken into account by the Belgian Committee of Medical Supply Planning who recently decided to progressively enlarge the production of physicians and to set minimal numbers for less attractive options. However, the lack of attractiveness of orientations such as general practice, child psychiatry and geriatrics does not find a satisfactory answer in formal numerical rules.

Second, geographical variations in head counts (but also in productivity) are noticed. To counterbalance the geographical imbalances, attraction policies have been recently implemented in order to attract but also to maintain physicians in underserved areas.

A last noteworthy phenomenon is the increase of foreigners in medical specialization and practice. This phenomenon gained momentum in recent years, generating questions concerning the planning of medical workforce supply that should be replaced in a broader perspective where physicians, but also patients, can migrate and do it effectively.

Countries included in the benchmarking exercise share a number of common challenges. Undoubtedly, cross-national comparisons offer an interesting tool for obtaining evidence on successfully developed and implemented initiatives in those countries.

### Shaping the future planning of medical workforce supply

The planning of the medical workforce supply involves determining the numbers, mix, and distribution of health providers that will be required to meet population health needs at some identified future point in time [34]. This paper has shown that it will be impossible to resolve the issues health policy planners are facing without taking a broader perspective on the health care system. Focusing on numbers is too restrictive. Adopting innovative policies learned from benchmarking without integration and coordination is unfruitful. Adopting a strategic planning is essential to control the effects of the complex factors impacting on human resources [35].

This is a complex task, implying three essential fields of activities:

1. An effective monitoring of all key factors affecting supply and demand;

2. A dynamic approach;

3. A system-level perspective [36].

### Effective monitoring of key factors to support decision-making

An in-depth evaluation of the current situation in human resources for health includes an assessment of the current stock of physicians and other healthcare workers; its composition, gender and age structure; its geographical distribution and its deployment between curative and preventive sectors but also between healthcare activities and other professional activities (teaching, research, administration, etc.); its activity profile (productivity levels) and working time; its forecasted evolution according to various scenarios; an analysis of the dynamics of the health labor market in terms of entries (including from national training and migration) and exits (deaths, age-related retirement, early retirement); the internal mobility between the public and the private sector, and between the different healthcare levels (primary care, general hospitals and highly specialized training hospitals). Sound policy development requires this type of data to ensure that policies are in line with the current and projected needs of health services.

Unfortunately, in most of the benchmarked countries, multiple datasets co-exist, but heterogeneous sources, collection strategies and parameters definitions lead to significant inconsistencies. These inconsistencies may even affect such crude measures as head counts of physicians and their translation into full-time equivalents, according to their productivity. The Netherlands addressed the problem, at least for data on GPs. Owing to cross-sectional surveys on sub-samples (e.g. the second Dutch National Survey of General Practice between 2000 and 2002), knowledge about demographical data and activity profiles of general practitioners was updated [37].

In general, data collection is poorly coordinated at the international level, and specifically in Europe. Due to the different organization and structure of the health care sector as well as the classification system used for health occupations and, finally, the policy priorities in each country, there is a strong variability of data among the various countries with deleterious consequences. Although new European regulations allow for the free movement of students and professionals, there is currently no good quality data to forecast, monitor and evaluate those international dynamics. There is also often a lack of specific data on health professionals. It is therefore not possible to develop a detailed pan-European or international picture of the migration trends of doctors, nurses and other health workers, or to assess the balance between temporary and permanent migrants [38]. Directorate General XV, Internal Market and Financial Services, collates statistics on the migration of doctors within the EU. Nevertheless, no data are available for many EU countries and available data are incomplete [39]. EUROSTAT Labor Force Survey reports the composition of foreign(-trained) physicians in selected OECD countries without mentioning the total number of immigrants [13]. As health professionals' shortages on one side of Europe may have an impact elsewhere, Europe-wide information is important for planning and providing health services for all health authorities throughout the EU [40].

Those issues can be addressed by:

• Improving and harmonizing definitions, guidelines and mechanisms used by international organizations for collecting data on the health workforce. The International Standard Classification of Occupations (ISCO) could be used as a reference, although additional categories and definitions of health workers are also needed. Ideally, these specifications should be agreed upon among international organizations [41].

• Coordinating and harmonizing routine data collection on the 'stock and flows' of medical supply. Data on head counts, actual level of activity, attrition or migration rate, should be validated and made readily available to stakeholders and researchers.

• Implementing complementary data collection for more specific information not provided routinely, such as practice arrangements, workload indicators or determinants of medical productivity. Regular surveying, both quantitative and qualitative, of a sub-sample of health care practitioners is an option.

• Identifying and monitoring indicators of health needs, such as disease trends or new clinical management, so as to allow a proper gap analysis.

• Setting up a national monitoring board accountable for providing policy-makers and stakeholders with yearly analysis of medical workforce. The National Observatory of Demography of Health Professions, in France, is an example of a body which gathers and analyzes data on medical demography, supports methodologically local and regional studies on that topic, and synthesizes and diffuses data and results.

### A dynamic approach

Medical supply planning needs to be sufficiently responsive and flexible to retain relevance and validity in a rapidly changing health system. There is no scientific means of assessing the appropriateness of manpower requirements. Instead, the definition of the adequacy of the medical manpower is a political competency and responsibility, reflecting broader societal decisions. In Belgium, the division of political healthcare responsibilities between federal, regional and community levels leads to a lack of a clear vision on the organization of health care provision. A profound political reflection is needed on the respective roles of different health service functions (hospitals, primary care, medical specialists, general practitioners, home nursing, etc.) and on how these functions connect within an overall vision on health services provision.

Those issues could be addressed by:

• Extending the global vision of healthcare delivery, taking into account additional parameters impacting on the medical supply (e.g. technology innovations, delivery of healthcare by other health professionals and by informal caregivers).

• Developing excellent linkages and exchanges among key stakeholders. Given the acknowledged limitations of the forecasting tools and multidisciplinary and collaborative networks, involving equally all stakeholders, is warranted [5].

### A system-level perspective

Medical supply planning is not only a matter of manpower size, but also encompasses the definition of the desired skill-mix, availability and accessibility level of medical services, quality control and accountability of health care providers, regulatory measures shaping the demand for health care, and financing of the health system. Without such system-level perspectives, medical supply planning takes the form of an exercise in demography based on implicit assumptions: that population age structure determines the service needs of the population and that the age and sex of providers determine the quantity of care provided [4].

Medical supply and requirement also depend upon professional boundaries. A lot of countries confronted with existing or emerging shortages of primary care physicians have adopted different solutions, including a re-defined role of the nursing workforce [42]. The availability of educational programs for advanced nursing practice (APN) is an important driver for the introduction of advanced nurse practitioners in a particular healthcare setting. Advanced nursing practice education at graduate level is currently offered in Europe in Belgium, Finland, Ireland, the Netherlands, Sweden, Switzerland and the United Kingdom [42].

Human resources for health policy would ideally help to define which types of workers - with what skills and in what quantity - will be needed, how they can be recruited, educated and trained over their professional lifetime, what working conditions and incentives can be offered to retain them and to motivate them to perform well, and how quality of practice would be monitored and ensured. Those choices should be validated by the various stakeholders to ensure a reasonable degree of feasibility in their implementation.

A number of challenges must be tackled in Belgium as in other selected European countries - in particular the lack of a comprehensive planning framework for different health professionals. Many policies are implemented that impact directly or indirectly on HRH without an adequate coordination or a formal evaluation scheme of such interventions.

Those challenges could be addressed by:

• Designing a national workforce planning framework. Examples of such a framework can be found in France in 2003 (ONDPS; http://www.ladocumentationfrancaise.fr/rapports-publics/064000455/index.shtml), Scotland in 2005 http://www.scotland.gov.uk/Publications/2005/09/20103932/39343 or in Australia in 2004 (AMWAC; http://www.nhwt.gov.au/documents/National%20Health%20Workforce%20Strategic%20Framework/Framework%20-%20Action%20Plan.pdf). Their main characteristics are being:

1. integrated (with all other planning systems, but particularly with service planning and resource/finance planning);

2. consistent and evidence-based (decisions are supported by sufficiently reliable information and robust methodologies);

3. with potential for evolution (flexible and adaptive to rapidly changing health system). Such frameworks define and diffuse the guiding principles of planning medical supply, and identify the actions that need to be taken at national or regional levels to tackle the challenges aforementioned.

• Setting up a body to design, monitor and evaluate the actions of the general planning framework.

• Creating realistic job descriptions for each type of healthcare worker and updating knowledge and competences needed to respond to new health problems. Linking training curricula to defined competences at all levels of healthcare workers' education may well foster the knowledge, skills and attitudes that health workers start with, and increase their potential for flexible and efficient learning in their continuing professional development later [41]. More dynamic and direct feedback channels from healthcare services or institutions to training schools and universities could help to bridge the gap between health demand and supply.

• Adopting successful initiatives from other countries may require significant adjustments in other sectors impacting health care systems (e.g. laws, financial regulations, labor market). Moreover, implementing new regulations should be considered as acceptable by the population or by professionals themselves. It is also paramount that such policy innovations be adequately evaluated.

## Conclusion

It is obvious that human resources for health policies that only focus on restricting the intake to the healthcare profession training without taking into account other factors related to evolving health needs and socio-demographic trends in workforce are likely to generate imbalances between the supply of and demand for health care labor. Ensuring an adequate, skilled and sustainable health workforce is clearly an urgent issue for health policy worldwide in order to face emerging changes related to demographic, technological, political, socioeconomic and epidemiological factors. While demand for health workers is increasing in many countries, health workforce planning remains a complex, difficult and not well understood process. To bridge the gap from 'trial and error' experience to evidence-based action, policy-makers need a global human resources for health picture, from their own country and abroad. As the efforts at the country level prove beneficial, human resources can be worked out at a more sustainable and reliable level.

## Abbreviations

AMWAC: Australian Medical Workforce Advisory Committee; APN: Advanced Nursing Practice; EEC: European Economic Community; EU: European Union: FTE: Full-Time Equivalent; GP: General Practitioner; HRH: Human Resources for Health; NP: Nurse Practitioner; OECD: Organisation for Economic Co-operation and Development; ONDPS: Observatoire National des Professions de Santé (France) [National Observatory for Health Professionals]; SP: Medical Specialist

## Competing interests

The authors declare that they have no competing interests.

## Authors' contributions

SS reviewed the literature and drafted the paper. CL critically reviewed and contributed to the writing. Both authors read and approved the final manuscript.
